# Cue duration determines response rate but not rate of acquisition of Pavlovian conditioning in mice

**DOI:** 10.1177/1747021820937696

**Published:** 2020-07-14

**Authors:** Joseph M Austen, David J Sanderson

**Affiliations:** Department of Psychology, Durham University, Durham, UK

**Keywords:** Learning, conditioning, mice, acquisition, reinforcement

## Abstract

The duration of a conditioned stimulus (CS) is a key determinant of Pavlovian conditioning. Rate estimation theory (RET) proposes that reinforcement rate is calculated over cumulative exposure to a cue and the reinforcement rate of a cue, relative to the background reinforcement rate, determines the speed of acquisition of conditioned responding. Consequently, RET predicts that shorter-duration cues require fewer trials to acquisition than longer-duration cues due to the difference in reinforcement rates. We tested this prediction by reanalysing the results of a previously published experiment. Mice received appetitive Pavlovian conditioning of magazine approach behaviour with a 10-s CS and a 40-s CS. Cue duration did not affect the rate at which responding emerged or the rate at which it peaked. The 10-s CS did elicit higher levels of responding than the 40-s CS. These results are not consistent with rate estimation theory. Instead, they are consistent with an associative analysis that assumes that asymptotic levels of responding reflect the balance between increments and decrements in associative strength across cumulative exposure to a cue.

The duration of a conditioned stimulus (CS) is an important determinant of conditioned responding in Pavlovian conditioning. It is commonly found that the rate of conditioned responding is greater for shorter-duration stimuli than longer-duration stimuli (Holland, 2000; Lattal, 1999). Real-time models of associative learning have incorporated several processes to account for the effect of cue duration, but they commonly assume that a CS will gain associative strength during periods of reinforcement and will lose associative strength during the periods of non-reinforced exposure prior to the onset of reinforcement within a trial (e.g., Brandon et al., 2003; Wagner, 1981). A long-duration CS has a greater period of non-reinforced exposure prior to reinforcement within a trial than a short-duration CS. Consequently, the loss of associative strength over the duration of the CS will be greater for a long-duration cue than a short-duration cue. All other things being equal, this difference in the balance between gains and losses of associative strength results in short-duration cues acquiring greater associative strength than long-duration cues.

A different account has been provided by rate estimation theory (RET) (Gallistel & Gibbon, 2000). In contrast to associative learning theories that assume that learning reflects the strengthening of associations between the memories of events, RET assumes that learning reflects the accumulation of evidence and that responding emerges when the evidence exceeds a decision threshold. It is assumed that the temporal properties of events are symbolically encoded. The cumulative duration of CS exposure and the cumulative number of reinforcements that a cue receives are encoded, and these variables are combined in order that animals may derive estimations of the rate of reinforcement. The rate of reinforcement during a CS relative to the background reinforcement rate determines the rate at which evidence accumulates and meets the decision threshold such that conditioned responding emerges. Because, in normal delay conditioning, animals never receive reinforcement in the absence of the CS, the estimate of the background reinforcement rate becomes increasingly low over training. In contrast, the reinforcement rate during the presence of the CS is constant. Consequently, the ratio of the CS reinforcement rate to the background reinforcement rate increases linearly over training. When the ratio of reinforcement rates exceeds a decision threshold, conditioned responding emerges. If the background reinforcement rate is held constant, then the shorter the duration of the CS, the greater the ratio of reinforcements rates and the faster the decision threshold is reached. Figure 1 depicts two hypothetical scenarios in which the ratio of reinforcement rates increases at different rates. This results in the decision threshold being reached in a differing number of trials.

**Figure 1.**
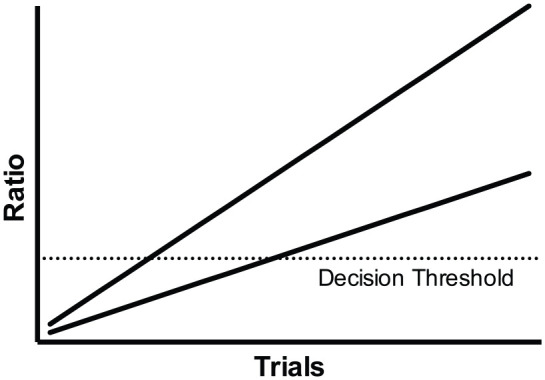
The underlying assumptions of rate estimation theory for acquisition. The ratio of the rate of reinforcement in the presence of the CS to the reinforcement rate of the background increases linearly over trials (as depicted by the two lines). In a continuous reinforcement procedure, the rate at which the ratio increases determines the number of trials required for the ratio to exceed a decision threshold. When the background reinforcement rate is held constant, the greater the rate of reinforcement during the CS, the faster the decision threshold will be reached. Thus, the steeper line reaches the decision threshold (dotted horizontal line) in fewer trials than the less steep line.

Although real-time associative accounts assume that short-duration CSs acquire greater associative strength than long-duration CSs, they are somewhat agnostic about the effect on the rate of acquisition unless specific assumptions are made about particular parameters. A real-time version of the Rescorla–Wagner model in which the learning rule is implemented iteratively over time assumes that different duration cues will reach different asymptotic levels of associative strength, but, due to the decrease in asymptote, long-duration cues will reach asymptote in fewer trials (see Figure 2). This effect is mediated by the relative difference between the excitatory and inhibitory learning rate parameters. It is typically assumed that the excitatory learning rate is greater than the inhibitory learning rate (Rescorla & Wagner, 1972). The extent of the relative difference between the learning rates reduces the extent of the advantage of long cues over short cues in trials to asymptote. Wagner’s (1981) Sometimes Opponent Process model (SOP model) captures many of the assumptions of the Rescorla–Wagner model, but, in addition, accounts for the effect of short-term habituation on associative learning. Wagner proposed that, as a consequence of short-term habituation, the salience of a cue diminishes within a trial such that a long-duration CS may, given particular parameters, be less able to form an association with an unconditioned stimulus (US) than a short-duration CS, reducing the speed of learning. Whether increases in cue duration reduce or increase the number of trials to asymptote likely depends on highly specific assumptions about particular parameters.

**Figure 2.**
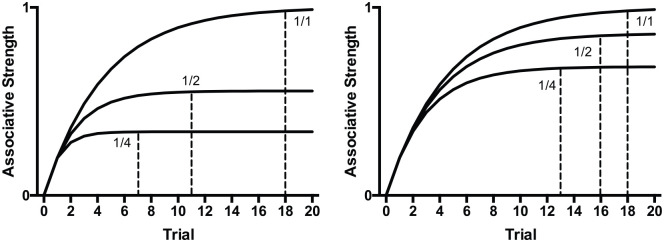
Simulations of the effect of CS duration on acquisition using the Rescorla–Wagner learning rule. Cue duration affects both asymptotic associative strength and rate of acquisition. Acquisition was simulated for CSs that were 1, 2 or 4 moments long. The 1-moment CS was reinforced every moment (1/1). The 2-moment CS was non-reinforced on the first moment and reinforced on the second moment of every trial (1/2). The 4-moment CS was non-reinforced on the first three moments and reinforced on the last moment of every trial (1/4). Lambda was set at 1. For the simulations in the left panel, the learning rate was set at 0.2 for both reinforced and non-reinforced moments. For the simulations in the right panel, the learning rate for reinforced moments was 0.2 and 0.04 for non-reinforced moments. The vertical dashed lines depict the trial at which subsequent increments in associative strength were less than 0.5% indicating that learning was close to the asymptotic level. Across both left and right panels, the number of trials to asymptote occurred within fewer trials the longer the duration of the CS. The difference in the trials to asymptote between the cues reduces as the ratio of the excitatory learning rate (the rate in the presence of the US) to the inhibitory learning rate (the rate in the absence of the US) increases. Thus, in the right panel in which the inhibitory learning rate was five times smaller than the excitatory learning rate, in addition to an increase in asymptote, the number of trials to asymptote was greater than compared to the left panel for the 1/2 and 1/4 cues.

Several studies have found that the rate of acquisition is faster with short-duration cues than long-duration cues (Bouton & Sunsay, 2003; Gibbon et al., 1977 but see also Kirkpatrick & Church, 2000). These studies have used between-subjects designs in which different groups of animals have been trained with different durations of cues. Although there may be differences in baseline responding that may affect measures of acquisition, it is also the case that between-group manipulations of cue duration inevitably confound either the intertrial interval between the offset of the CS and onset of the next CS presentation by controlling the US–US interval or the US–US interval by controlling the intertrial interval (Gibbon et al., 1977). It is possible, given the proposed roles of reinforcement rate and short-term habituation, that the intertrial interval and the US–US interval may have independent effects on response rates. This issue can be avoided by using a within-subjects design in which a common intertrial interval is used. Another issue is that these studies have measured acquisition by calculating the number of trials taken to reach a response threshold based on an absolute measure of responding. As noted by Kirkpatrick and Church (2000), a response threshold based on an absolute measure of responding confounds the measure of acquisition with measures of the strength of responding. For example, responding may be acquired at the same rate under two conditions and may reach asymptotic levels at the same rate but responding may reach the criterion sooner under one condition than the other because of achieving a higher asymptotic level of responding. This issue can be avoided by using a threshold based on a relative rather than an absolute measure of responding.

The purpose of the present study was to test whether cue duration does affect the rate at which conditioned responding emerges in a manner that avoids the issues discussed above. This was tested in appetitive conditioning of magazine approach behaviour in mice. We analysed the trials to acquisition of a data set previously reported by Austen and Sanderson (2019). In Experiment 1 of that study, we reported the group-level response rates to a short- and long-duration cue over sessions of acquisition. It was found that the short-duration cue came to elicit higher asymptotic levels of conditioned responding than the long-duration cue. It is not possible to tell from that analysis, however, whether acquisition was faster for the short-duration cue compared to the long-duration cue.

In the Austen and Sanderson (2019) study, two cues that differed in duration were reinforced at the termination of the cue in a standard delay conditioning procedure. For half of the mice, the short- and long-duration cues were a fixed 10- and 40-s duration, respectively. For the other half, the cues varied in duration trial by trial, but had mean durations that were the same as for the mice that were trained with fixed cue durations. The duration of cues varied uniformly around the mean durations such that responding could not be timed to an expected delay of reinforcement (Harris et al., 2011). It has been argued that response rates are more directly related to reinforcement rates when responses cannot be timed to the occurrence of reinforcement (Harris et al., 2015). By comparing acquisition across mice trained with fixed- and variable-duration cues, it provides a way of assessing the role of reinforcement rate as manipulated by cue duration under circumstances in which timing of responding is more or less likely to influence performance.

Although RET (Gallistel & Gibbon, 2000) proposes that the rate of acquisition is reflected by the number of trials to the emergence of responding, associative theories generally assume that the rate of learning is reflected by the number of trials to asymptote (e.g., Rescorla & Wagner, 1972). This is based on the assumption that the acquisition of learning reflects a negatively accelerating curve. The largest change in responding should occur at the start of training with subsequent changes becoming smaller as learning progresses. Consequently, the number of trials to the emergence of responding will not differentiate between the rate of acquisition of cues, but the speed at which responding reaches its maximum level will. The key difference between these accounts is whether it is assumed that acquisition is abrupt with the maximum rates of responding achieved as soon as responding emerges or whether acquisition is a gradual incremental process. Gallistel et al. (2004) concluded that the typically observed negatively accelerating learning curve is an artefact of group averaging and individual animals show abrupt changes in response rates. In contrast, others (e.g., Harris, 2011; Jennings et al., 2013) have observed that there is often, but not always, a delay between the emergence of responding and asymptotic response rates suggesting that acquisition is a gradual process. Because of the different assumptions about how the rate of acquisition is determined, we examined both trials to the emergence of responding and trials to peak responding.

## Method

### Subjects

Thirty-two naïve female C57BL/6J mice (Charles River UK Ltd), approximately 10 weeks old at the start of testing, with a mean free-feeding weight of 19.1 g (range: 15.9–22.6 g), were used. Mice were caged in groups of 4–8 in a temperature-controlled housing room on a 12-hr light–dark cycle (lights on at 8:00 am). Prior to the start of the experiment, the weights of the mice were reduced by being placed on a restricted diet. Mice were then maintained at 85% of their free-feeding weights throughout the experiment. Mice had ad libitum access to water in their home cages. All procedures were conducted under Home Office UK project license number PPL 70/7785 and approved by the local Animal Welfare Ethical Review Board.

### Apparatus

A set of eight identical operant chambers (interior dimensions: 15.9 cm × 14.0 cm × 12.7 cm; ENV-307A, Med Associates, Inc., Fairfax, VT, USA), enclosed in sound-attenuating cubicles (ENV-022V) were used. The operant chambers were controlled by Med-PC IV software (SOF-735). The side walls were made from aluminium, and the front and back walls and the ceiling were made from clear Perspex. The chamber floors each comprised a grid of stainless steel rods (0.32 cm diameter), spaced 0.79 cm apart, and running perpendicular to the front of the chamber (ENV-307A-GFW). A food magazine (2.9 cm × 2.5 cm × 1.9 cm; ENV-303M) was situated in the centre of one of the sidewalls of the chamber, into which sucrose pellets (14 mg, TestDiet) could be delivered from a pellet dispenser (ENV-203-14P). An infrared beam (ENV-303HDA) across the entrance of the magazine was used to record head entries at a resolution of 0.1 s. A fan (ENV-025F) was located within each of the sound-attenuating cubicles and was turned on during sessions, providing a background sound pressure level of approximately 65 dB. Auditory stimuli were provided by a white noise generator (ENV-325SM) outputting a flat frequency response from 10 to 25,000 Hz at 75 dB and a clicker (ENV-335M) operating at a frequency of 4 Hz at 75 dB. Visual stimuli were a 2.8 W house light (ENV-315M) which could illuminate the entire chamber, and two LEDs (ENV-321M) positioned to the left and right of the food magazine, which provided more localised illumination.

### Procedure

Mice received 12 sessions of training with two short-duration cues and two long-duration cues. Mice were randomly allocated to one of two groups (*N* = 16 per group). For group fixed, the duration of short cues was 10 s and the duration of long cues was 40 s. For group variable, the durations of the cues varied from trial to trial, but within a session they had a mean duration that was the same as the duration of group fixed. Therefore, the short cues had a mean of 10 s, but the duration of each trial varied, according to a uniform distribution, around the mean (shortest = 2 s and longest = 18 s). Similarly, the long cues had a mean of 40 s, and trials varied according to a uniform distribution around the mean (shortest = 2 s and longest = 78 s). For both groups, one of the short- and one of the long-duration cues was reinforced by the presentation of a sucrose pellet at the termination of the cue (CS+). The remaining short and long cues were non-reinforced (CS−). Within each group, for half of the mice, the short cues were auditory (noise, clicker) and the long cues were visual (house light, flashing LEDs (0.25 s on/0.25 s off)). The opposite was true for the remaining mice. Within each of these subgroups, the identity of the reinforced and non-reinforced stimuli was fully counterbalanced. Each of the four cues was presented nine times per session with a fixed interval of 120 s between the offset of one cue and the onset of the next. Trials were presented in a random order with the constraint that an equal number of each cue was presented every block of 12 trials. For each session, all mice received the stimuli presented in the same order (e.g., first trial = noise, second trial = house light, third trial = clicker). Due to the identity of short- and long-duration cues and the identity of reinforced and non-reinforced cues being counterbalanced across mice, this resulted in the order of these factors also being counterbalanced across mice.

### Data analysis

The analyses focused on the acquisition of responding with the two reinforced cues. Therefore, analyses of the non-reinforced cues are not reported. We first followed the method of Gallistel et al. (2004) of fitting Weibull cumulative distributions to the trial-by-trial response rates (number of magazine entries, rate per minute) for each mouse. Rates of responding were converted to differences scores by subtracting the rate of responding during the 10-s pre-CS period from the rate of responding during the CS for each trial. The three parameters for determining the Weibull function can be used to determine the emergence of responding, the asymptotic response rate and the number of trials from the emergence of responding to asymptotic levels of responding. We do not report these results, however, because a proportion of mice failed to show patterns of responding that matched a sigmoidal function that could be characterised by a cumulative Weibull distribution. A further problem that arose was that, for some mice, the Weibull distribution fitted an asymptotic level of responding that was not achieved by the mouse within the total number of trials. When applied to the acquisition of responding in rats, the trial at which responding reaches 10% of asymptote has been used as a measure of latency to acquisition (Harris, 2011; Jennings et al., 2013). Because the measure of the emergence of responding is dependent on the asymptotic response rate as determined by the fitted Weibull distribution, some mice never achieved this relative level of performance. The combination of these two issues meant meaningful parameters could not be calculated for approximately a sixth of the mice for the 10-s cue and approximately a third of the mice for the 40-s cue.

Given the shortcomings of trying to fit Weibull distributions to the data, we decided instead to make some simpler assumptions about the data to derive measures of acquisition. We chose a response criterion for acquisition based on a measure of performance that was relative to each mouse’s baseline level of responding. The trial at which responding was reliably above baseline for each mouse was assumed to be the point at which conditioned responding emerged. To determine the point at which conditioned responding emerged above baseline for each cue, we first calculated difference scores, in which pre-CS response rates were subtracted from CS response rates for each trial. The mean of the pre-CS response rates for the 10- and 40-s reinforced cues per trial was used to compare the two cues to a common baseline. The cumulative difference score across trials for each cue was then calculated. By plotting the cumulative difference score, it is possible to observe where responding remains at the baseline level by the cumulative record being relatively flat across trials. The point at which responding emerges above baseline results in an upward trajectory in the cumulative record across trials. We identified the point at which responding emerged above baseline by locating the trial at which there was an upward trend that was sustained over a number of trials. Although this was often the point at which the cumulative difference became consistently positive, for some mice the cumulative difference score would initially decrease below zero (due to suppression of responding) before there was a positive increase. Therefore, it was necessary to determine the point at which there was an initial positive increase in the cumulative difference score rather than the point at which the score became positive. A positive slope in the cumulative difference score was determined by calculating linear trends for each consecutive block of six trials (i.e., trials 1–6, 2–7). This method was chosen rather than comparing the cumulative difference score trial by trial because it reduced the influence of trial-by-trial fluctuations in response rates. To determine if there was a consistent positive trend in the cumulative difference score, a criterion of six consecutive trial blocks with a positive slope was used. The trial at which the consecutive run of positive slopes began was considered to be the point at which the acquisition of responding occurred. For example, if the slopes for trial blocks 1–6, 2–7, 3–8, 4–9, 5–10, and 6–11 were all positive, then the number of trials to acquisition was 6. Additional analyses were conducted (but not reported here) in which a more stringent criterion of 10 consecutive trials with a positive slope was used. This produced a similar pattern of results and, in the vast majority of cases, the number of the trial in which the criterion was met was the same as when the six consecutive trials criterion was used.^1^

The peak response rate that was achieved for each cue was determined by calculating a running average of the trial-by-trial difference scores over six trials (i.e., trials 1–6, 2–7). These running averages reduced the effect of trial-by-trial variation in response rates. The peak response rate that was achieved after the trial at which the acquisition criterion was met was recorded for each cue and also the trial at which it was first achieved.

Data were subjected to 2 (cue: 10 and 40 s) by 2 (group: fixed and variable) analysis of variance (ANOVA). The modality of the 10-s cue was also included as a nuisance factor. We have previously found that mice respond more to auditory cues than visual cues (Sanderson et al., 2016). Therefore, the variance caused by this counterbalancing factor was controlled by including it in the ANOVA, but the main effect of modality and any interactions that included modality were ignored. In addition to the ANOVA, we report Bayes factors (BF_10_) for the effect of cue for each measure. These were calculated using Bayesian repeated-measures ANOVA in JASP (Love et al., 2019) using the default priors (Cauchy scale set to 0.707). For consistency, we uniformly report Bayes factors for all analyses, but the Bayesian statistics were run to assess the level of evidence for the null hypothesis in instances in which non-significant results were found. All analyses were run in JASP.

In addition to the measures of acquisition, we wished to address whether differences in response rates to the two cues reflected the effect of cue duration on learning or on the performance of responding. Responding to cues across trials, within sessions, confounds the effect of cue duration on performance and learning. Within a session, after the first trial with each cue, the short- and long-duration cues differ in the duration of recent experience. Therefore, subsequent trials do not assess performance under comparable test conditions for each cue. Furthermore, a comparison of performance across trials that differ in duration means that performance is not measured across a comparable time. To match the test conditions for each cue, such that the effect of cue on learning could be dissociated from an effect on performance, we assessed responding to the first 10 s of the short- and long-duration cues on the first trial of each trial type of the sessions in the latter half of training (sessions 7–12) when response rates for the two cues had diverged (Austen & Sanderson, 2019). The average pre-CS response rates across trial types were subtracted from responding to the short- and long-duration cues to derive a difference score compared to a common baseline. Although it is possible that performance during the first 10 s may reflect differences between the cues that occur as a result of timing reinforcement, this is unlikely to be the case for the variable group. Although the average delay of reinforcement differs for the two cues for the variable group, the variability of the duration of the cues ensures that reinforcement cannot be timed. Indeed, reinforcement was equally likely at different time points between 2 and 18 s for the variable 10-s cue and 2–78 s for the variable 40-s cue. Therefore, for the variable group, a comparison of the response rates during the first 10 s of the initial trials within a session of the short- and long-duration cues provides a measure of learning that is independent of an effect of cue duration on performance and independent of the influence of timing. It is important to note that the initial trials of a session for short- and long-duration cues for the variable group were all at least 10 s in duration for sessions 7–12. This occurred as a consequence of the random selection of the variable intervals rather than by design. In addition, the trial order of short- and long-duration cues was counterbalanced across mice within a session. The data were subjected to the same analyses as for the other measures of acquisition and responding.

## Results

**Figure 3.**
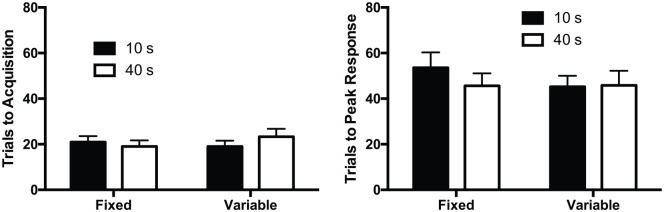
Rate of acquisition for the fixed and variable cue duration groups. Left panel: the mean number of trials to acquisition criterion for the 10- and 40-s cues. Right panel: the mean number of trials to the peak response rate. Error bars indicate SEM.

The number of trials to acquisition is shown in Figure 3, left panel. The trials to acquisition were similar for the 10- and 40-s cues, and there was little difference between the fixed and variable groups. The effect of cue was not significant, *F*(1,28) < 1, *p* = .61. There was no significant effect of group, *F* < 1, *p* = .70, and no significant interaction of factors, *F*(1,28) = 1.76, *p* = .20. The Bayesian analysis of the effect of cue revealed that BF_10_ = 0.27, suggesting that the results provided 3.5 times more evidence for the null hypothesis than the alternative hypothesis^2^.

The number of trials to peak response rate is shown in Figure 3, right panel. The trials to peak response rate were similar for the 10- and 40-s cues and there was little difference between the fixed and variable groups. The effect of cue was not significant, *F*(1,28) < 1, *p* = .58. There was no significant effect of group, *F* < 1, *p* = .60, and no significant interaction of factors, *F*(1,28) < 1, *p* = .37. The Bayesian analysis of the effect of cue revealed that BF_10_ = 0.30, suggesting that the results provided 3.3 times more evidence for the null hypothesis than the alternative hypothesis.

**Figure 4.**
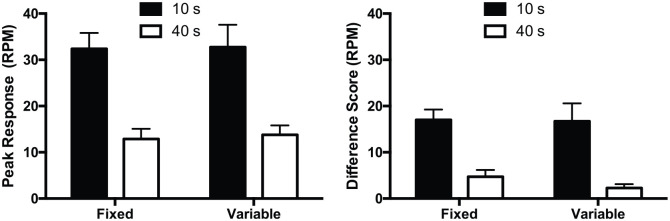
Response rates per minute (RPM) for the fixed and variable cue duration groups. Left panel: mean peak response rates for the 10- and 40-s cues. Right panel: mean response rates under matched test conditions for the two cues. Error bars indicate SEM.

The peak response rate for each cue is shown in Figure 4, left panel. Mice showed higher peak response rates for the 10-s cue than for the 40-s cue. This was true for both groups. The effect of cue was significant, *F*(1,28) = 139.94, *p* < .001. There was no significant effect of group, *F* < 1, *p* = .72, or interaction of factors, (*F* < 1, *p* = .88). The Bayesian analysis of the effect of cue revealed that BF_10_ > 10^5^.

The rates of responding during the first trials of each trial type of each session for sessions 7–12, restricted to the first 10 s, are shown in Figure 4, right panel. Mice responded more to the 10-s cue than the 40-s cue. This was true for both groups. The effect of cue was significant, *F*(1,28) = 96.54, *p* < .001. There was no significant effect of group, *F*(1,28) = 1.00, *p* = .33, or interaction of factors, *F* < 1, *p* = .44. Bayesian analysis of the effect of cue revealed that BF_10_ > 10^5^.

We conducted additional analyses of the response rates for the first trial of each session for group fixed. In contrast to group variable, for group fixed responses could be timed to the occurrence of reinforcement, and consequently response rates may change over the course of the trial. The analysis reported above found a significant difference between the response rates in the first 10 s of the two cues. It is possible, however, that response rates may be similar over the whole duration (10 and 40 s) of the cues. This was not the case and mice responded significantly more for the 10-s cue than for the 40-s cue across the whole duration of the cues, (10-s cue mean response rate per minute minus pre-CS response rate = 17.03, ±2.23 SEM; 40-s cue mean response rate per minute minus pre-CS response rate = 6.08, ±1.35 SEM; *F*(1,14) = 26.62, *p* < .001; BF_10_ = 174). It is also possible that although the response rates differ over the duration of the 10- and 40-s cues, the response rates close to the time of reinforcement may be similar. Thus, responding to the 40-s cue may start at a low rate but rise to a similar level as for the 10-s cue. To test this possibility responding over the whole duration of the 10-s cue was compared to the last 10 s of the 40-s cue (i.e., over comparable periods of time prior to the time of reinforcement). Mice responded significantly more to the 10-s cue (mean response rate per minute minus pre-CS response rate = 17.03 ± 2.23 SEM) than to the 40-s cue (mean response rate per minute minus pre-CS response rate = 7.03 ± 1.60 SEM; *F*(14) = 21.34, *p* < .001; BF_10_ = 47).

## General discussion

RET (Gallistel & Gibbon, 2000) proposes that cues that differ in reinforcement rate will differ in the number of trials to acquisition. We tested this prediction by training mice with two cues that differ in duration such that the short-duration cue had a higher reinforcement rate than the long-duration cue. The results failed to demonstrate a significant difference in the number of trials to reach an acquisition criterion for each cue, and both the fixed and variable duration groups showed a similar pattern of results. The Bayesian analysis of the effect of cue demonstrated evidence in favour of the null hypothesis. Similarly, there was no significant difference in the number of trials to reach asymptote. Therefore, regardless of how the rate of acquisition was measured, there was no significant difference between the cues.

The different duration cues did elicit different levels of responding. This was true for measures of peak response rates and also for the analysis of responding under matched test conditions for the short- and long-duration cues. The latter result demonstrates that the difference in responding to the cues is not due to the effect of cue duration on performance but due to an effect on learning. Thus, although responding emerged at similar time points and reached asymptote at similar time points for the two cues, the actual extent of acquisition was different for the short- and long-duration cues.

As stated above, we dissociated an effect on learning from a performance effect by matching the test conditions for the two different cues. Under matched test conditions any differences in responding to the different cues must reflect prior learning about the cues rather than the influence of the cue manipulation on performance of responding during the test of learning. This approach has been used by others (e.g., Holland, 2000). An alternative approach to test the strength of learning, independent from performance effects, has been to assess the ability of the cues to restrict learning with other cues (Bonardi et al., 2015; Jennings & Bonardi, 2017). For example, if cues differ in their strength of learning, then they should differ in their ability to block the acquisition or expression of conditioned responding with new cues. This indirect way of assessing learning may be less prone to ceiling and floor effects that may occur. Indeed, we have previously found that cues that elicit similar levels of conditioned responding may result in different levels of blocking (Sanderson et al., 2016). A potential disadvantage of this method, however, is that cue competition effects may be mediated by the strength of within-compound associations between the competing cues (Blaisdell et al., 1999). Within-compound associations depend on the associability of the cues. If cue competition depends on within-compound associations, then effects such as blocking do not provide a pure measure of learning. For example, two cues may have an equal strength of learning, but differ in their associability. This would result potentially in the cues eliciting different levels of blocking. Although it is clear that there are advantages and disadvantages to the use of any single method for the assessment of learning independent of performance effects, our approach of matching the test conditions provides a test based on the simple assumption that there is an ordinal relationship between the strength of responding and learning.

In the test of learning under matched test conditions, we compared responding during the first 10 s of the two cues. This method compares responding across the entirety of the 10-s cue and the first quarter of the 40-s cue. Given that animals may time responding, it could be argued that it is preferable to compare responding across the whole duration of the two cues. Response rates may be particularly low in the first 10 s of the 40-s cue compared to the 10-s cue because mice withhold responding until the latter parts of the cue closer to the time of reinforcement. This is not likely to be the case, however, for the mice in the variable group. For these mice, reinforcement could not be timed because the time of reinforcement varied across trials. Indeed, we found, in our previous analysis, that the gradients of the distribution of responding over time within trials were shallower for the variable group than the fixed group (Austen & Sanderson, 2019). Despite the differences in the timing of responding between the two groups, both groups showed a similar advantage of the 10-s cue over 40-s cue in the strength of response rates. This suggests that the observed cue duration effect under matched test conditions did not primarily reflect timing of responding.

Given the potential, however, that timing may affect response rates for the fixed group, we conducted additional analyses comparing response rates across the whole duration of the cues and during equivalent periods prior to reinforcement. It was still found that mice responded at a higher rate for the 10-s cue than for the 40 s cue. Therefore, it is unlikely that the difference in response rates for the fixed group was primarily due to differences in timing of conditioned responding.

There was no significant effect of cue duration variability on any of the measures. This is somewhat consistent with other studies that have examined the effect of cue duration variability on the acquisition of conditioned responding (Jennings et al., 2013; Ward et al., 2012), although Jennings et al. (2013) found that fixed-duration cues elicited higher asymptotic response rates and were slower to acquire asymptotic rates of responding. Our results fail to support the proposal that informativeness in terms of temporal certainty affects rates of acquisition (Balsam & Gallistel, 2009).

Although the pattern of results does not fit with accounts of learning that assume that the reinforcement rate or cue duration affects the rate of learning, they are, instead, consistent with associative accounts that assume reinforcement rate affects the asymptotic level of responding. For example, if an error correction rule is implemented iteratively over cumulative exposure to a cue, then during periods of reinforcement associative strength will increase, but during periods of non-reinforcement associative strength will decrease. The ratio of increments to decrements in associative strength over cumulative exposure results in associative strength reflecting the cumulative reinforcement rate of a cue.

The current results fail to support the hypothesis that the reinforcement rate affects the number of trials to acquisition. Gallistel and Gibbon (2000), however, suggested that data from other species and conditioning procedures are consistent with that hypothesis. For example, they cite a study by Gibbon et al. (1977) that reports that trial duration affects the number of trials to acquisition in a pigeon autoshaping procedure. As discussed in the “Introduction” section, it is important to note that Gibbon et al. (1977) used a criterion for acquisition based on an absolute measure of responding (three out of four trials with at least one response). The problem with an absolute measure is that animals that obtain different asymptotic levels of responding are likely to reach the criterion at different speeds even when they do not differ in the speed at which responding initially emerges (see Kirkpatrick & Church, 2000, for a discussion). Therefore, it is not clear whether the effect of trial duration on pigeon autoshaping reflects an effect on the rate of acquisition, or, instead, absolute strength of responding, which is readily explained by an associative account.

For RET (Gallistel & Gibbon, 2000), identifying the factors that determine the rate of acquisition is crucial for understanding learning. The model assumes that learning reflects the accrual of evidence to determine when to start responding. Once responding emerges, the strength of responding simply reflects performance factors rather than the underlying learning processes. Associative theories, in contrast, assume that the strength of responding reflects the strength of the learning. The rate of acquisition provides information about the associability or salience/intensity of the events that are being learnt about, but does not provide information about the strength of learning (e.g., Mackintosh, 1975; Pearce & Hall, 1980; Rescorla & Wagner, 1972). The current results are in line with an associative analysis and not with RET. Nevertheless, the results do not falsify the underlying assumptions that RET makes about the mechanisms of learning. There are other ways in which to disentangle the two opposing theories such as examining whether learning reflects a step function, consistent with evidence reaching a decision threshold, or a negatively accelerating curve, consistent with an associative analysis (Gallistel et al., 2004; Glautier, 2013; Harris, 2011; Kehoe et al., 2008; Morris & Bouton, 2006). Before these theoretical issues are resolved, it may be fruitful to re-examine the properties of events and the specific parameters that determine the rate of acquisition and extent of conditioning to identify the type of information that would be relevant for decision making accounts of conditioned responding.

## Notes

1. For the mice for which it was possible to derive meaningful parameters from the Weibull distribution, there was a positive correlation between the emergence of responding parameter and the trials to criterion measure for the 10-s cue (*r* = .52, *p* = .006, *N* = 27). This was also true for the 40-s cue, but the correlation was not significant (*r* = .37, *p* = .086, *N* = 22). Therefore, there is a high degree of correspondence between the two methods. This was also true for the peak response measure and the asymptote parameter from the Weibull distribution (for both cues, *r* > .91, *p* < .001).

2. The distribution of data was somewhat positively skewed with a few mice acquiring responding after a large number of trials. However, the difference between the 10-s and 40-s cues for each mouse was not skewed. Analyses of the data converted to natural log values yielded a similar pattern of conclusions as found with the analysis of the raw scores.
